# A Continuous Non-Invasive Blood Pressure Prediction Method Based on Deep Sparse Residual U-Net Combined with Improved Squeeze and Excitation Skip Connections

**DOI:** 10.3390/s24092721

**Published:** 2024-04-24

**Authors:** Kaixuan Lai, Xusheng Wang, Congjun Cao

**Affiliations:** 1 The Faculty of Printing, Packaging Engineering and Digital Media Technology, Xi’an University of Technology, Xi’an 710048, China; 2220820042@stu.xaut.edu.cn (K.L.); xswang@xaut.edu.cn (X.W.); 2 The Printing and Packaging Engineering Technology Research Center of Shaanxi Province, Xi’an 710048, China

**Keywords:** continuous blood pressure prediction, photoplethysmography, U-net, sparse residual connections, SE-GRU, temporal features, deep supervision

## Abstract

Arterial blood pressure (ABP) serves as a pivotal clinical metric in cardiovascular health assessments, with the precise forecasting of continuous blood pressure assuming a critical role in both preventing and treating cardiovascular diseases. This study proposes a novel continuous non-invasive blood pressure prediction model, DSRUnet, based on deep sparse residual U-net combined with improved SE skip connections, which aim to enhance the accuracy of using photoplethysmography (PPG) signals for continuous blood pressure prediction. The model first introduces a sparse residual connection approach for path contraction and expansion, facilitating richer information fusion and feature expansion to better capture subtle variations in the original PPG signals, thereby enhancing the network’s representational capacity and predictive performance and mitigating potential degradation in the network performance. Furthermore, an enhanced SE-GRU module was embedded in the skip connections to model and weight global information using an attention mechanism, capturing the temporal features of the PPG pulse signals through GRU layers to improve the quality of the transferred feature information and reduce redundant feature learning. Finally, a deep supervision mechanism was incorporated into the decoder module to guide the lower-level network to learn effective feature representations, alleviating the problem of gradient vanishing and facilitating effective training of the network. The proposed DSRUnet model was trained and tested on the publicly available UCI-BP dataset, with the average absolute errors for predicting systolic blood pressure (SBP), diastolic blood pressure (DBP), and mean blood pressure (MBP) being 3.36 ± 6.61 mmHg, 2.35 ± 4.54 mmHg, and 2.21 ± 4.36 mmHg, respectively, meeting the standards set by the Association for the Advancement of Medical Instrumentation (AAMI), and achieving Grade A according to the British Hypertension Society (BHS) Standard for SBP and DBP predictions. Through ablation experiments and comparisons with other state-of-the-art methods, the effectiveness of DSRUnet in blood pressure prediction tasks, particularly for SBP, which generally yields poor prediction results, was significantly higher. The experimental results demonstrate that the DSRUnet model can accurately utilize PPG signals for real-time continuous blood pressure prediction and obtain high-quality and high-precision blood pressure prediction waveforms. Due to its non-invasiveness, continuity, and clinical relevance, the model may have significant implications for clinical applications in hospitals and research on wearable devices in daily life.

## 1. Introduction

With the continuous acceleration of global population aging and urbanization, the incidence of cardiovascular diseases (CVDs) is steadily increasing, gradually becoming some of the diseases with the highest incidence and mortality rates [1]. According to the relevant reports, cardiovascular diseases accounts for the largest proportion of disease-related deaths in both rural and urban residents, with CVDs accounting for 48.00% and 45.86% of deaths in rural and urban areas, respectively, in 2020 [2]. Despite the high level of attention given to the prevention and control of cardiovascular diseases, the upward trend in their incidence has not been fundamentally reversed, making cardiovascular diseases a major global public health issue [3].

Blood pressure (BP) is a crucial physiological indicator of the human circulatory system, comprising systolic blood pressure (SBP) and diastolic blood pressure (DBP) [4]. Monitoring these metrics aids in evaluating an individual’s blood pressure status. Hypertension represents a significant risk factor for cardiovascular ailments, encompassing heart disease, stroke, arteriosclerosis, and various other cardiovascular complications. According to the inaugural Global Hypertension Report unveiled by the World Health Organization in 2023 [5,6], the global prevalence of hypertension surged from 650 million in 1990 to 1.3 billion in 2019. Consequently, the quest for achieving portable, continuous monitoring of human blood pressure to facilitate early detection, prevention, and treatment of hypertension and cardiovascular diseases has emerged as a paramount concern.

Currently, blood pressure measurement methods mainly include direct measurement, intermittent blood pressure measurement, and continuous non-invasive blood pressure measurement [7,8,9]. Direct blood pressure measurement involves inserting a catheter into the artery to directly monitor real-time blood pressure data. While this method is unaffected by external noise, its invasive nature poses risks of infection and arterial damage. Intermittent blood pressure measurement methods commonly used include the auscultatory method and the oscillometric method. The auscultatory method [10] utilizes a stethoscope to listen to blood flow sounds to determine the systolic and diastolic pressures. Although convenient, it is subject to subjective errors and may lead to the “white coat” phenomenon. Conversely, the oscillometric method [11] automatically measures blood pressure using oscillations beneath the blood pressure cuff, offering convenience and practicality. However, the measurement method based on cuff inflation and deflation repetitively compresses the arterial blood vessels, leading to psychological discomfort and other issues. As a result, it cannot continuously track dynamic blood pressure changes and achieve accurate continuous blood pressure measurement.

In the realm of continuous blood pressure monitoring, methods based on pulse transit time (PTT) and pulse wave velocity (PWV) calculate arterial blood pressure by measuring the time or velocity required for a pulse to travel from one location to another. However, both these methods require frequent calibration to actual blood pressure values, posing limitations in terms of measurement accuracy and applicability [12,13]. Additionally, these methods typically necessitate at least two fully synchronized input signals (such as PPG and ECG signals) to obtain accurate physiological parameters. Ensuring strict synchronization of ECG and PPG signals in time during PTT measurements, as well as ensuring that the R peak of the ECG signal corresponds to the main peak of the PPG signal within the same cardiac cycle, significantly increases the complexity of blood pressure prediction tasks and the amount of raw data required, making them less suitable for clinical research [14,15,16].

In addition to utilizing pulse wave parameters for blood pressure prediction, many researchers have explored the relationship between various physiological signals and blood pressure through the construction of mathematical models. For instance, Shi et al. [17] combined electrical network models with tube-load models to propose a hybrid mathematical model for establishing the relationship between PPG signals and blood pressure signals. Through system identification methods, individualized continuous blood pressure measurements can be achieved. Similarly, Yi et al. [13] established the relationship between piezoelectric pulse waves and blood pressure waves using linear and integral relationships, enabling wearable continuous blood pressure prediction without motion artifacts. These approaches, based on specific assumptions and inferences, offer strong predictive performance and interpretability by elucidating the relationship between blood pressure changes and PPG signal features. However, acquiring medical physiological datasets that encompass various physiological states is often challenging. This limitation extends to parameter tuning for the established mathematical models.

The continuous advancement of deep learning has provided new perspectives for continuous blood pressure prediction, offering an end-to-end learning paradigm that can directly learn the mapping relationship between input and output from raw data [18]. Traditional methods require the manual extraction of physiological parameters and features from input signals, often involving complex feature engineering and data preprocessing steps, making them unsuitable for efficient and precise wearable products [19,20]. Deep learning models possess high-performance feature extraction capabilities and the ability to handle large-scale data, enabling them to capture individual differences and blood pressure variations without the need for complex physical modeling. Moreover, they can automatically tune parameters, laying the technical foundation for achieving accurate and continuous blood pressure monitoring [21]. With its formidable feature extraction and information mining capabilities, deep learning has been widely employed in the field of continuous non-invasive blood pressure prediction based on PPG signals. For instance, Baek et al. [22] utilized Convolutional Neural Networks (CNNs) with dilated and strided convolutions in both the time and frequency domains to extract features from periodic signals, achieving accurate blood pressure prediction. Sadrawi et al. [23] employed deep convolutional autoencoders based on LeNet and U-Net architectures to transform PPG signals into ABP signals. Schrumpf et al. [16] trained blood pressure prediction models based on PPG signals using three different deep learning models, combined with signal parameterization methods for empirical evaluation. They further fine-tuned the network models using transfer learning to successfully apply them to clinical environments for blood pressure prediction based on rPPG signals. Numerous studies [24,25,26] have demonstrated a high degree of similarity between PPG and blood pressure waveforms, highlighting the significance of recovering original blood pressure waveforms for clinical research. Therefore, in addition to predicting blood pressure parameters, this study also attempted to reconstruct the original blood pressure waveform using only a single PPG signal, revealing the patterns of blood pressure changes. 

Since the proposal of the U-shaped architecture (U-net) by Ronneberger et al. [27], this model has garnered significant attention from scholars due to its highly symmetric structure and the paradigm of skip connections, and has been widely applied in the field of blood pressure prediction. Cheng et al. [28] constructed ABP-Net for blood pressure waveform prediction through the design of the network structure, input signals, and loss functions. It allows for non-invasive estimation of physiological parameters reflecting the cardiovascular status, albeit with room for improvement in accuracy. Athaya et al. [25] introduced new activation functions and dropout optimization to enhance the traditional U-net structure, demonstrating its potential for blood pressure prediction and potential application in sensor-based wearable devices. Ibtehaz et al. [26] developed a dual-layer U-net model comprising an approximate network and a refinement network, achieving the precise prediction of blood pressure waveforms but falling short of meeting the A-grade criteria of the BHS standard in the systolic blood pressure prediction task. Sun et al. [29] proposed a dual-channel encoder U-net model and incorporated an improved attention mechanism block into the encoder to address the strong periodicity and continuity characteristics of PPG signals, thereby achieving accurate and rapid blood pressure prediction.

However, existing research indicates that there is still room for improvement in using the U-net model for continuous blood pressure prediction. Firstly, the direct transmission of long-distance information via skip connections for high–low-scale feature fusion may lead to information redundancy and loss. Additionally, the use of ordinary convolutions for information transmission in the upsampling and downsampling paths may result in information loss and gradient vanishing issues [30]. Finally, the traditional U-net primarily focuses on extracting and reconstructing local features, which presents certain limitations in capturing global contextual information. However, physiological signals such as PPG signals exhibit strong temporal and continuous characteristics, posing challenges for U-net in effectively extracting their temporal features.

In response to the aforementioned issues, this paper proposes a novel continuous non-invasive blood pressure prediction method based on deep sparse residual U-net combined with improved SE skip connections, aiming to enable continuous blood pressure prediction using a single PPG signal. The main contributions of this work are as follows:

(1) The introduction of a highly symmetric DSRUnet architecture, incorporating refined sparse residual connections to facilitate feature propagation, thereby enhancing information fusion and feature expansion for capturing subtle variations in PPG signals. To address the issue of the inability of the fully connected layers in the SE module to dynamically learn temporal data features, GRU layers are introduced to capture temporal pulse signal features by learning internal channel dependencies. Furthermore, an SE-GRU module is embedded within the skip connections for global information modeling and weighting, aimed at enhancing the discriminative and representational capabilities of essential features in the original PPG signal.
(2) The integration of a deep supervision mechanism by introducing additional output layers at the decoder end of the DSRUnet network, guiding the lower-level network to learn effective feature representations, thus alleviating the issue of gradient disappearance and improving the network’s training efficiency and performance.
(3) The proposed method not only predicts highly accurate SBP, DBP, and MBP values but also enables the accurate recovery of blood pressure waveforms from a single PPG signal. Extensive ablation experiments and comparisons with the existing research demonstrate the superior blood pressure prediction performance of the DSRUnet model proposed in this study, particularly in SBP prediction, surpassing other state-of-the-art models in terms of accuracy, thus indicating its potential applicability in wearable devices.

The remaining sections of the paper are organized as follows: Section 2 provides a detailed description of the research methodology for continuous non-invasive blood pressure prediction based on photoplethysmography (PPG) signals. It also outlines the fundamental and innovative theories behind the proposed DSRUnet model. Section 3 encompasses the experimental settings, dataset descriptions, establishment of evaluation metrics, and configuration of the comparative models. Section 4 elucidates the experimental results and analysis, including assessments based on various standards, results from ablation experiments, and comparisons with existing methods. Section 5 summarizes the research findings of the paper and outlines potential future research directions.

## 2. Materials and Methods

**Figure 1:**
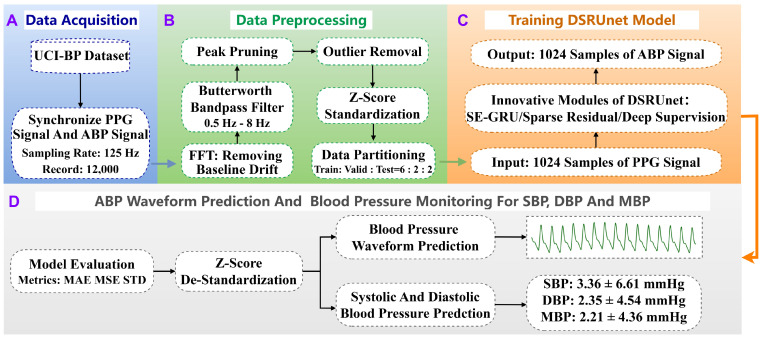
Overall research framework for continuous non-invasive blood pressure prediction based on PPG signals. The process consists of four stages: (**A**) data acquisition, (**B**) data preprocessing, (**C**) model training, and (**D**) model validation and prediction. Stage (**D**) includes both blood pressure waveform prediction and blood pressure parameter prediction.

To enhance the generalization and representational capacity of the blood pressure prediction network and address limitations in information fusion, global feature modeling, and gradient vanishing, we propose a Deep Sparse Residual U-net (DSRUnet) for continuous non-invasive blood pressure prediction. The network employs U-net as its core framework, comprising traditional encoder–decoder modules and skip connections, and incorporates structural optimization strategies such as sparse residuals, SE-GRU, and deep supervision. This section primarily describes the research methodology for continuous non-invasive blood pressure prediction based on photoplethysmography (PPG) signals, including data acquisition, preprocessing, model training, and prediction. The specific research framework is illustrated in Figure 1. Additionally, this section elaborates on the basic theory and innovative modules of the proposed DSRUnet model, sequentially introducing these modules and analyzing their roles and innovations in the blood pressure prediction task.

### 2.1. Blood Pressure Prediction Task Based on PPG Signals

The blood pressure prediction task based on photoplethysmography (PPG) signals aims to accurately predict individual blood pressure values by leveraging the temporal and spectral features of PPG signals, in conjunction with deep learning or machine learning models [31]. PPG signals, acquired through non-invasive optical sensors, represent variations in skin microvascular blood flow induced by heartbeats, which are closely associated with cardiac activity and vascular status [32]. By analyzing and mining the feature information of PPG signals, the blood pressure prediction task elucidates the relationship between PPG features and blood pressure-related parameters, providing non-invasive, real-time means of blood pressure monitoring for medical and health care, with significant clinical application prospects.

The main blood pressure parameters include SBP, DBP, and MBP. In blood pressure signals, SBP represents the highest pressure point, typically corresponding to the maximum value during cardiac contraction, while DBP represents the lowest pressure point, typically corresponding to the maximum value during cardiac relaxation. These points can be identified in the blood pressure waveform by monitoring the peaks and troughs of the blood pressure signal [33]. Mean blood pressure (MBP) is also a significant physiological parameter in blood pressure monitoring. It reflects the average arterial pressure level throughout the cardiac cycle and aids in assessing blood pressure regulation function [34]. It should be noted that the values of SBP, DBP, and MBP are calculated and may vary depending on changes in physiological conditions.

Based on relevant mathematical knowledge, this paper defines the blood pressure prediction task based on PPG signals as a regression task aimed at minimizing the target loss function. Let X represent the original input PPG signal and Y represent the corresponding blood pressure signal. The task objective is to learn an optimal mapping function f:X→Y, which accurately transforms the PPG signal into the blood pressure signal. This mapping function can be represented by Equation (1).

(1) $$Y=f\left(X\right),$$

In this process, the numerical values of the regression model’s parameters and the relationship between the final PPG signal and blood pressure signal are determined through training a deep learning model. The optimal mapping function $f^{*}\left(x\right)$ can be obtained by minimizing the objective loss function, as shown in Equation (2).

(2) $$f^{*}\left(X\right)=\arg\min\left(L\left(f\left(X\right),Y\right)\right),$$

In this context, $L\left(\cdot ,\cdot \right)$ represents the loss function. 

Therefore, given the original inputs, the predicted SBP, DBP, and MBP can be computed using Equations (3)–(5).

(3) $$SBP=\max\left(f^{*}\left(X\right)\right),$$

(4) $$DBP=\min\left(f^{*}\left(X\right)\right),$$

(5) $$MBP=\frac{1}{3}\left(SBP+2DBP\right),$$

### 2.2. Overall Framework of DSRUnet Network

**Figure 2:**
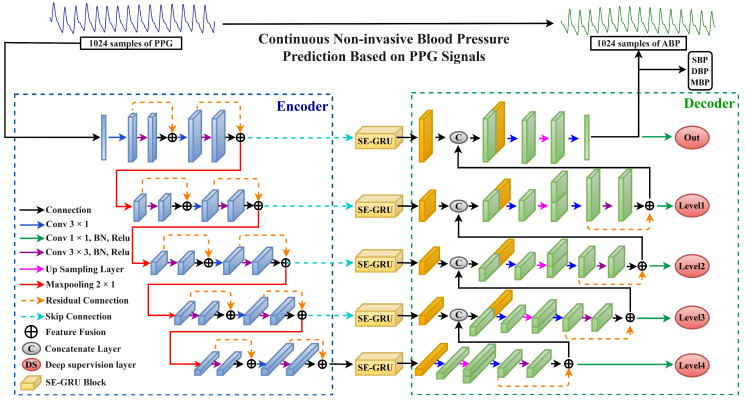
The overall structure of the proposed DSRUnet network model is depicted, with the blue dashed box representing the encoder part, and the green dashed box representing the decoder part.

This section provides a detailed overview of the overall framework of the proposed DSRUnet network model. It adopts the conventional U-net network architecture, consisting of encoder and decoder modules, each comprising four downsampling modules and four upsampling modules, respectively. Both the downsampling and upsampling paths consist of multiple sparse residual connection modules, with corresponding dimensional skip connections linked by SE-GRU modules. The SE attention module facilitates the transmission of features learned in the encoder to the corresponding decoder, assisting the decoder in recovering detailed information. The improved GRU module enables the model to adapt more effectively to the characteristics of temporal pulse data, enhancing the network’s perception and utilization efficiency of important features. Furthermore, deep supervision was introduced into the network by introducing supervisory signals at different levels of the model’s output, allowing the model to learn and optimize from multiple levels, thereby accelerating convergence, improving robustness, and mitigating the issue of gradient vanishing. The overall framework of the network model is illustrated in Figure 2.

### 2.3. SE-GRU Module

#### 2.3.1. Original SE Attention Mechanism Module

**Figure 3:**
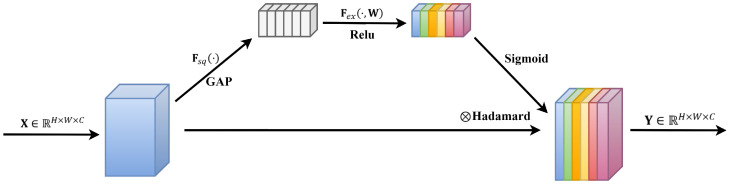
Original SE attention mechanism module structure.

The SE (Squeeze-and-Excitation) attention mechanism, a technique employed to enhance the representational capability of features, was initially proposed by Hu et al. [35] in 2018. This mechanism adjusts the importance of each feature channel by learning feature weights, thereby increasing the model’s focus on important features. The SE module primarily consists of two steps: Squeeze and Excitation. The Squeeze step involves global average pooling, converting the feature maps of each channel into global features for each channel, compressing the feature maps along the spatial dimension to obtain a global feature description for each feature channel. Subsequently, in the Excitation step, weights for each channel are learned using fully connected layers, which are then applied to weight the feature channels to obtain enhanced feature representation. The framework schematic of the original SE attention mechanism module is illustrated in Figure 3.

Assuming the input features are denoted as $X\in {\mathbb{R}}^{H\times W\times C}$, where H, W, and C represent the height, width, and number of channels, respectively, the operations of the SE module can be described in the following three steps.

(1) Squeeze Operation: Perform a Squeeze operation on X, utilizing global average pooling to map each H×W matrix of X into a global feature channel descriptor, $z_{C}\in {\mathbb{R}}^{C}$, as shown in Equation (6).

(6) $$z_{C}=F_{sq}\left(X\right)=\frac{1}{W\times H}\sum_{i=1}^{W}\sum_{j=1}^{H}X\left(i,j\right),$$

(2) Excitation Operation: Conduct an Excitation operation on X by learning channel-specific activation weights ω through a linear layer, followed by a Sigmoid function to obtain distinct excitation weights s, as depicted in Equation (7).

(7) $$s=F_{ex}\left(z,W\right)=\sigma \left(g\left(z,W\right)\right)=\sigma \left(W_{2}\delta \left(W_{1}z\right)\right),$$

where δ represents the Relu activation function; $W_{1}\in {\mathbb{R}}^{C/r\times C}$ and $W_{2}\in {\mathbb{R}}^{C\times C/r}$ are the weight parameters of the fully connected layer; r represents the scaling factor; and in this model, σ signifies the Sigmoid activation function.

(3) Apply the excitation weights s to each channel of the input features X to obtain the final enhanced feature output Y, as shown in Equation (8).

(8) Y=X⊗s,

where ⊗ represents element-wise multiplication (Hadamard product), and $Y\in {\mathbb{R}}^{H\times W\times C}$ denotes the feature output after being processed by the SE module.

#### 2.3.2. Improved SE Attention Mechanism Module

In the original SE module, the global channel descriptor obtained after compression is conveyed through a fully connected layer. However, fully connected layers are typically utilized for processing flattened input data and are unable to capture dependencies between sequential features [36]. Their fixed parameter relationships imply an inability to model temporal dependencies within data and to dynamically learn relationships between features. Moreover, the considerable parameter count not only increases model complexity but also tends to lead to overfitting, thereby compromising the model’s generalization ability. As a result, they are unsuitable for utilizing temporal pulse signals for blood pressure prediction.

**Figure 4:**
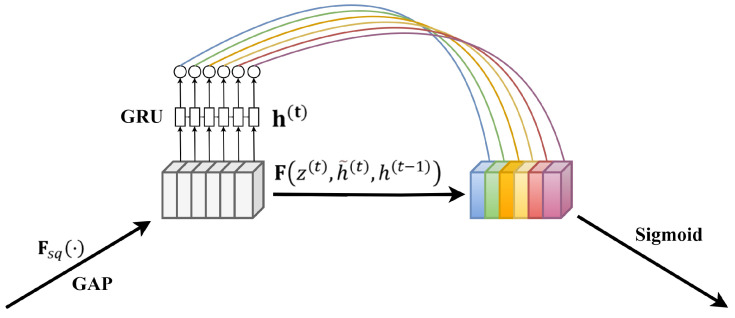
Schematic diagram of SE-GRU module structure. The GRU layer is mainly used to replace the fully connected layer to capture the timing pulse characteristics.

Therefore, this paper proposes an improvement to the original SE module’s approach of obtaining internal channel dependencies using fully connected layers, aiming at the temporal characteristics of PPG signals and blood pressure signals. We introduced a GRU layer capable of capturing temporal data features, thus presenting a more suitable SE-GRU module for blood pressure prediction utilizing temporal pulse signals. Initially, the module compresses the original features into global channel descriptors via the Squeeze operation. Subsequently, by inputting the global channel descriptors into the corresponding GRU layer, the module leverages the gate mechanism within the GRU units to dynamically adjust the current hidden state based on the current input and the previous hidden state. This process facilitates the acquisition of output weights for different channels, enabling more effective learning and representation of dynamic features and dependencies within sequential data. Finally, the obtained output weights are used to weight the original features, restoring them to their original dimensions. The proposed SE-GRU module is illustrated in Figure 4.

GRU, a variant of recurrent neural networks [37], is designed for handling sequential data and possesses inherent memory capabilities. It comprises two gate units: the Reset Gate and the Update Gate. The Reset Gate controls the degree of retention of past information, while the Update Gate regulates the degree of integration of new information.

Assuming at time step t, the input is $x^{\left(t\right)}$ and the hidden state is $h^{\left(t-1\right)}$, the computation of the Reset Gate $r^{\left(t\right)}$ and the Update Gate $z^{\left(t\right)}$ in the GRU is formulated as shown in Equations (9) and (10), respectively.

(9) $$r^{\left(t\right)}=\sigma \left(W_{r}\cdot \left[h^{\left(t-1\right)},x^{\left(t\right)}\right]+b_{r}\right),$$

(10) $$z^{\left(t\right)}=\sigma \left(W_{z}\cdot \left[h^{\left(t-1\right)},x^{\left(t\right)}\right]+b_{z}\right),$$

Here, σ denotes the Sigmoid function, and $W_{r}$, $W_{z}$, $b_{r}$, and $b_{z}$ represent the weight parameters. In the application of the Reset Gate, new memory content utilizes the Reset Gate to store relevant past information. The computation of the candidate hidden state ${\tilde{h}}^{\left(t\right)}$ is formulated as shown in Equation (11).

(11) $${\tilde{h}}^{\left(t\right)}=\tanh\left(W\cdot \left[r^{\left(t\right)}⊙h^{\left(t-1\right)},x^{\left(t\right)}\right]+b\right),$$

Here, ⊙ represents the Hadamard product, and W and b are weight parameters. The final memory computation process requires the use of the Update Gate, which determines the current memory content and the information to be gathered from the previous time step. The update of the hidden state is formulated as shown in Equation (12).

(12) $$h^{\left(t\right)}=\left(1-z^{\left(t\right)}\right)⊙h^{\left(t-1\right)}+z^{\left(t\right)}⊙{\tilde{h}}^{\left(t\right)},$$

Embedding the SE-GRU module into the skip-connection path facilitates a more accurate transmission of features learned in the encoder to the corresponding decoder segments, enhancing feature representation and emphasizing critical details such as peaks, valleys, and waveform shapes. The fundamental concept is to dynamically weight the features transmitted through the skip connections to highlight important feature information relevant to the current task, thereby improving the network’s perception and utilization efficiency of essential features. Specifically, feature information generated through convolutional operations is input into the SE-GRU module, comprising global average pooling and a GRU layer. Through global average pooling, the feature information of each channel is transformed into the corresponding global features. Subsequently, the GRU layer learns temporal pulse features, obtaining weights for each channel, which are then used to weight the feature channels, resulting in enhanced feature representation. Finally, the features processed by the SE module are transmitted to the corresponding decoder network structure, aiding in the recovery of detailed information by the decoder and supporting subsequent feature extraction and learning, thus enhancing the model’s predictive performance and generalization capability.

### 2.4. Sparse Residual Connection Module

**Figure 5:**
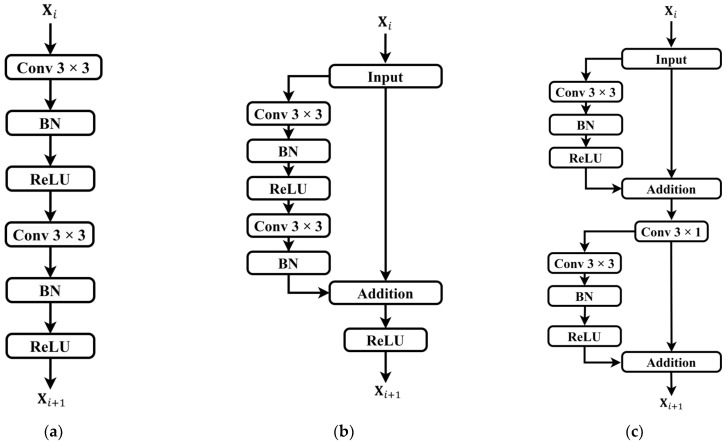
Comparison of convolutional units and residual units. (**a**) Ordinary convolution unit; (**b**) ordinary residual unit; (**c**) sparse residual unit. It should be noted that only partial modules of the sparse residual units are utilized in the upsampling pathway.

While attention mechanisms assist base networks in extracting salient features from input signals, deep models encounter challenges such as gradient vanishing and performance degradation with increasing convolutional layers [38]. Residual connection [39] is a technique used in deep neural networks to address issues of vanishing and exploding gradients. Its core idea involves introducing direct skip connections between certain layers of the network, allowing information to flow directly from lower to higher layers. This facilitates easier learning of residuals, i.e., the differences between the target output and the current predicted output, thereby enhancing model training effectiveness and convergence speed. The ordinary convolutional unit and residual unit are illustrated in Figure 5a,b.

Each residual convolutional unit can be represented by Equations (13) and (14).

(13) $$y_{i}=F\left(x_{i},W_{i}\right)+h\left(x_{i}\right),$$

(14) $$x_{i+1}=f\left(y_{i}\right),$$

where $x_{i}$ and $x_{i+1}$ are the input and output of the residual unit, $F\left(\cdot \right)$ is the residual function, $H\left(\cdot \right)$ is the identity mapping function, and $f\left(\cdot \right)$ is the activation function.

In order to better extract features from the raw PPG time-series data and assist the model in adapting to complex data distributions and task requirements, an improvement was made to the conventional residual units by introducing a sparse residual connection approach. In the encoder module, each input feature undergoes a residual connection after only one convolutional layer and a batch normalization (BN) layer. Subsequently, the obtained feature’s original information is concatenated to generate the first residual information. Then, the feature undergoes another round of processing by the convolutional layer and BN layer to obtain the second residual information. This transformation converts a single residual connection in the original residual unit into two sparse residual connections, while the corresponding decoder path adopts a sparse residual connection only once. Replacing traditional convolution operations in the contraction and expansion paths with this sparse residual connection approach allows for the direct transmission of input information to the output, alleviating potential issues of gradient transmission hindrance commonly associated with conventional residual connections. This approach enables more effective capturing and utilization of the input data’s feature information, and deeper network structures are no longer constrained by gradient vanishing issues. The proposed sparse residual connection module, after improvement, is depicted in Figure 5c.

### 2.5. Deep Supervision Module

Deep supervision is a method for training deep neural networks [40,41], which involves adding additional auxiliary outputs in the middle layers of the network to provide more supervision signals. These outputs offer supervision at different depths of the network to aid faster convergence and better learning of feature representations, thereby enhancing the understanding and prediction capabilities using blood pressure signals. The model’s auxiliary outputs at each level enable the capture and prediction of blood pressure changes at different scales. Original blood pressure signals may be affected by noise such as motion interference and signal drift. Deep supervision, through additional supervision signals, allows the network to learn more robust feature representations, enhancing the model’s resistance to noise and improving the training efficiency and accuracy. In this study, five deep supervision layers were added, placed individually after the five outputs in the decoder path, denoted as the “out” layer, and “level1” to “level4” layers. The loss weights for each layer were set as [1, 0.9, 0.8, 0.7, 0.6], respectively. The loss function is shown in Equation (15).

(15) $$L_{total}=\sum_{i=1}^{N}\left(L\left(y,p_{out}\right)+\sum_{j=1}^{4}L\left(p_{level_{j}}\right)\right),$$

where $p_{out}$ is the final output prediction, $p_{level_{j}}$ is the prediction of the j auxiliary output, and N is the number of samples.

## 3. Experimental Settings

### 3.1. Experimental Environment and Parameter Settings

The deep learning framework employed in this study is TensorFlow 2.13.0, which was run on the Windows 10 operating system. The GPU utilized was NVIDIA GeForce RTX 2080 with 8GB dedicated GPU memory. The algorithm implementation and experimental validation were conducted using Python 3.8. The proposed blood pressure prediction network was trained using the Adam optimizer, with a batch size set to 256. Each experiment was run for 100 epochs, incorporating an early stopping mechanism. Specifically, if the performance on the validation set did not improve continuously for 10 consecutive epochs, the training process was immediately halted. The learning rate of the network was set to 0.0001.

To effectively evaluate the model’s performance, the mean absolute error (MAE) was employed as the loss function. As per related research [42], MAE demonstrates better robustness in the presence of motion artifacts and noise, and by balancing all error terms, it showcases superior performance. The calculation of MAE is depicted in Equation (18) below. Additionally, the Mean Squared Error (MSE) was used as an additional metric to monitor the model, providing a better assessment of the difference between the predicted results and the actual data, as shown in Equation (19) in Section 2.3.

### 3.2. Experimental Dataset

#### 3.2.1. UCI-BP Dataset

To train and evaluate the proposed DSRUnet network, the UCI-BP dataset provided by the University of California Irvine (UCI) machine learning repository was utilized. This dataset, compiled by Kachuee et al. [43,44], was sourced from the MIMIC-II database and comprises synchronized continuous fingertip PPG signals, ABP signals, and ECG signals from 12,000 records of ICU patients. Each record has a duration ranging from 8 to 592 s, with a sampling frequency of 125 Hz for all signals. The precision of the recordings is 8 bits. The dataset is stored in four .mat files, labeled as Part_1 to Part_4, each containing 3000 cell arrays. Each cell represents a record, and each row of the record corresponds to a signal channel. This study specifically utilized the synchronized PPG and ABP signals. A statistical summary of the UCI-BP dataset from [26] is presented in Table 1.

It can be observed that SBP had a significantly larger standard deviation value. This indicates that when using this dataset for blood pressure prediction, predicting the SBP parameter may result in larger errors, which aligns with the hypothesis proposed by Kachuee et al. [44]. Therefore, in the final evaluation of the model’s predictive performance, for networks with similar performance, this study determined the optimal blood pressure prediction model based on the accuracy and effectiveness of predicting the SBP parameter, as proposed by the DSRUnet network.

#### 3.2.2. Data Preprocessing

In the task of blood pressure prediction, high-quality data are essential for the model to learn pulse and blood pressure features effectively. Reasonable data preprocessing methods can improve data quality and reliability [45]. Therefore, this study referred to previous research [22,24,46,47] and performed preprocessing operations on the PPG signals and blood pressure signals in the UCI-BP dataset, including baseline drift removal, bandpass filtering, outlier removal, data partitioning, and data standardization. Firstly, records with a time span less than 8 min were removed, reducing the total number of records from 12,000 to 2064, ensuring the reliability of the final PPG and blood pressure signals [48,49]. Baseline drift in the original PPG signals was removed using Fourier Transform (FFT). Subsequently, a fourth-order Butterworth bandpass filter with a low cutoff frequency of 0.5 Hz and a high cutoff frequency of 8 Hz, corresponding to the sampling frequency of 125 Hz, was applied to eliminate low-frequency and high-frequency noise present in the PPG and blood pressure signals. 

To eliminate abnormal peak values in the signals, the peak clipping method [50] was employed for correction and adjustment. In the first step, the mean and standard deviation were calculated to determine the threshold. Linear interpolation was then applied in the left and right regions of the abnormal peaks based on the difference between the signal value and the threshold, gradually approaching the set threshold. This effectively eliminates peak abnormalities and fluctuations, resulting in smoother and more reliable signals. 

To eliminate inter-individual differences and differences in scale among different features, the Z-score standardization method [51] was applied to the original PPG signals for data standardization. The specific method is illustrated by Equation (16).

(16) $$z=\frac{x-\mu }{\sigma },$$

Here, x represents the original PPG signal data, μ denotes the mean of the original data, σ represents the standard deviation of the original data, and z signifies the standardized PPG signal data. By using the Z-score for data standardization, data with similar scales and distributions are obtained, making the feature weights learned by the model more generalizable and enhancing the model’s generalization ability. The statistical data of the UCI-BP dataset after data preprocessing are presented in Table 2, where it can be observed that the standard deviation values of each parameter have decreased, which facilitates subsequent model training for prediction. 

**Figure 6:**
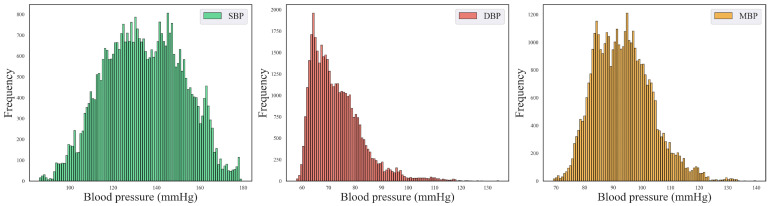
Blood pressure distribution plots for SBP, DBP, and MBP.

The model training in this study was ultimately divided into training, validation, and test sets in a ratio of 6:2:2. The training set comprised 23,648 samples, while the validation and test sets each contained 7808 samples, and the length of each sample was 1024. The blood pressure data consisted of three channels representing SBP, DBP, and MBP. The final distribution of the blood pressure information is illustrated in Figure 6.

### 3.3. Model Evaluation Metrics

This study adopted several evaluation metrics to assess the blood pressure prediction model’s performance. These metrics include mean error (ME), mean absolute error (MAE), Mean Squared Error (MSE), standard deviation (STD), and Coefficient of Determination (R-squared) [52]. The specific calculation method is shown in Equations (17)–(21).

(17) $$ME=\frac{1}{N}\sum_{i=1}^{N}\left(y_{i}-{\hat{y}}_{i}\right),$$

(18) $$MAE=\frac{1}{N}\sum_{i=1}^{N}\left|y_{i}-{\hat{y}}_{i}\right|,$$

(19) $$MSE=\frac{1}{N}\sum_{i=1}^{N}{\left(y_{i}-{\hat{y}}_{i}\right)}^{2},$$

(20) $$STD=\sqrt{\frac{1}{N}\sum_{i=1}^{N}{\left(y_{i}-{\hat{y}}_{i}\right)}^{2}},$$

(21) $$R^{2}=1-\frac{{\sum_{i=1}^{N}\left(y_{i}-{\hat{y}}_{i}\right)}^{2}}{\sum_{i=1}^{N}{\left(y_{i}-\bar{y_{i}}\right)}^{2}},$$

where N presents the number of samples, $y_{i}$ represents the true blood pressure value, ${\hat{y}}_{i}$ represents the predicted blood pressure value, and ${\bar{y}}_{i}$ represents the mean of the true values. These metrics provide comprehensive insights into the accuracy, precision, and reliability of the blood pressure prediction model.

### 3.4. Comparative Model Settings

To validate the roles of various modules in the proposed DSRUnet model and facilitate subsequent ablation experiments on the preprocessed UCI-BP dataset, seven networks were established for the ablation experiments and comparative validation based on the traditional U-net network’s encoder–decoder structure. These networks were set up with different modules at different positions in the network architecture, as shown in Table 3. They included the proposed DSRUnet network, where each network utilizes deep supervision. The skip connection methods were set to the original SE modules and SE-GRU modules, while the transmission methods for the upsampling and downsampling paths were set to the regular residual and sparse residual. Model 1 does not include any additional modules, representing the simplest U-net structure.

## 4. Results and Discussion

### 4.1. Evaluation and Analysis of Experimental Results Based on BHS Standard

The British Hypertension Society (BHS) standard [53] is one of the international standards used to assess the accuracy of blood pressure measurement devices. It serves as a foundation for determining whether blood pressure prediction models can be applied in clinical experiments. The accuracy criteria in the BHS standard evaluation method are established based on absolute errors, requiring evaluation based on the percentage of absolute errors in the predicted values for the test samples. The thresholds are set at 5 mmHg, 10 mmHg, and 15 mmHg, and the BHS defines three grades, A, B, and C, as shown in Table 4. The overall evaluation grade for the SBP, DBP, and MBP predictions under the three scenarios was determined using the worst result among the three sets of threshold judgment results.

**Figure 7:**
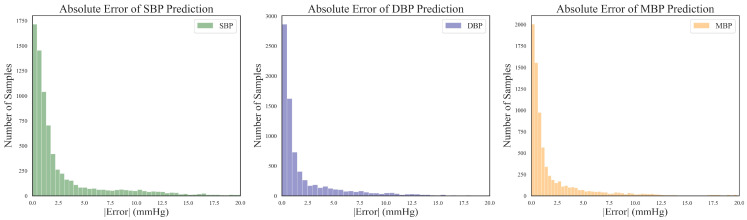
Distribution of absolute errors for SBP, DBP, and MBP predictions.

Utilizing the BHS standard, the evaluation grade results for the different models are presented in Table 5. It can be observed that the proposed DSRUnet model achieved grade A in the evaluation of its SBP, DBP, and MBP predictions according to the BHS standard. Furthermore, compared with the other models, the DSRUnet model achieved significant breakthroughs in the prediction accuracy for SBP after incorporating the improved SE-GRU module and sparse residual connection module. Specifically, the percentage of predictions below a difference of 5 mmHg exceeded 80% for the first time, and predictions below 10 mmHg exceeded 90%. Although the performance of this model in DBP and MBP prediction was slightly lower than that of Model 5 and Model 6, comparing DSRUnet with Model 6 revealed that the prediction accuracy of SBP was enhanced by 1.51%, 0.75%, and 0.34% under the three thresholds, respectively. Meanwhile, the prediction accuracy of DBP decreased by only 0.08%, 0.45%, and 0.14%, respectively. This suggests that the improvement in SBP prediction performance by DSRUnet far outweighed the decrease in DBP prediction performance. Considering the substantial difficulty in predicting SBP in blood pressure prediction tasks, this outcome is acceptable according to the hypothesis proposed by Kachuee et al. [44,54]. Figure 7 illustrates the distribution of absolute errors when predicting SBP, DBP, and MBP. It can be observed that the majority of absolute errors were below 2.5 mmHg, indicating that the proposed model achieved small errors in blood pressure prediction and exhibits good prediction performance, meeting the basic requirements for clinical applications [55].

### 4.2. Evaluation and Analysis of Experimental Results Based on AAMI Standard

Similar to the BHS standard, the Association for the Advancement of Medical Instrumentation (AAMI) Standard [56] serves as another benchmark for assessing the performance of medical devices. In the realm of blood pressure measurements, the AAMI standard is frequently employed to gauge the accuracy and reliability of blood pressure measuring devices. This standard imposes specific requirements regarding error limits, stipulating that the average error and standard deviation between predicted and true results should each be less than 5 mmHg and 8 mmHg, respectively. Furthermore, adherence to the AAMI standards typically necessitates a minimum sample size of 85 subjects in the study. Table 6 presents the evaluation results of the DSRUnet model in accordance with the AAMI standard.

From the evaluation results, it is evident that the proposed DSRUnet model meets the AAMI standard for blood pressure prediction. Building upon this foundation, to assist researchers and medical professionals in assessing the consistency between predicted and true results, Bland–Altman plots [57] of the predicted SBP, DBP, and MBP results are generated based on the ME and STD evaluation metrics. These plots provide a visual means to evaluate potential biases or anomalies in the predicted results and reflect the central tendency of the predictions. Bland–Altman plots utilize the standard deviation of the differences to describe the variability of the mean. The requirement for good consistency between true and predicted results is that the vast majority of differences fall within the 95% limits of agreement, defined as ±1.96 times the standard deviation of the differences. The range of this limit is $\left[\mu -1.96\sigma ,\mu +1.96\sigma \right]$, where μ and σ represent the mean and standard deviation of the differences, respectively. This range can reflect the acceptable level in clinical practice.

**Figure 8:**
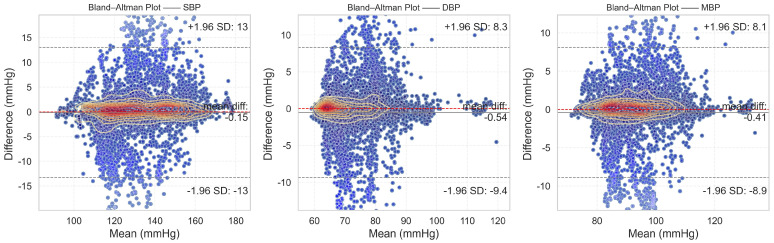
Bland–Altman plots of SBP, DBP, and MBP prediction results. Different shades of transparency in the colors represent the distance from the mean error baseline: the closer the scatter points are to the baseline, the darker the color, and the farther away from the baseline, the more transparent the color.

The final results, as shown in Figure 8, distinctly illustrate the consistency analysis of the SBP, DBP, and MBP predictions using the DSRUnet model. The majority of the errors were below 5 mmHg, with the local density heatmaps distributed near the 0 scale line. Although some samples exceeded 15 mmHg in the SBP predictions, the distribution of the local density heatmap in this scenario was the most uniform. This demonstrates that the DSRUnet model has relatively reliable performance for blood pressure prediction, particularly with a qualitative breakthrough in SBP predictions. Moreover, the results can be validated through consistency checks.

### 4.3. Ablation Experiment and Result Analysis

In order to comprehensively evaluate the generalization ability and predictive performance of the proposed DSRUnet model, degradation experiments were conducted based on the model evaluation metrics set in Section 2.3 and the comparative models set in Section 2.4, using an independent test set. The evaluation results of the different models are presented in Table 7. To facilitate a more intuitive comparison of the ME metric differences, the absolute values of the ME results were used for comparison.

**Figure 9:**
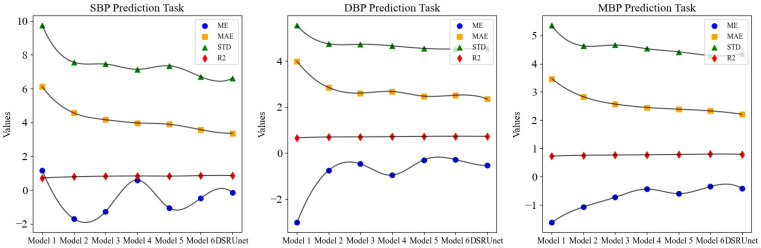
Evaluation index prediction results of different models for different blood pressure parameter predictions.

The comparison of four performance metrics across different models is illustrated in Figure 9. It is evident that the DSRUnet model exhibited superior performance, indicating its advanced capabilities.

From the results of the ablation experiment, it can be observed that the performance the pure U-net network without any improvement modules for continuous blood pressure prediction was the poorest. In particular, the MAE for SBP predictions reached 6.11 and the STD reached 9.73, indicating very unsatisfactory results. Building upon the performance of the worst-performing Model 1, different improvement modules were progressively added for the degradation experiment.

A comprehensive comparison and analysis of the experimental results of each model, using the mean absolute error (MAE) of each prediction as the evaluation metric, validates the effectiveness of the proposed innovative modules for SBP, DBP, and MBP predictions. The specific results are as follows:

- Model 2 and Model 3 have embedded traditional SE modules and improved SE-GRU modules, respectively, in the skip-connection part. Compared to Model 1, the MAE for SBP predictions was reduced by 1.55 and 1.95, respectively, for Model 2 and Model 3. Similarly, for DBP prediction, the MAE was reduced by 1.14 and 1.38, respectively. This demonstrates that improving the skip-connection method can significantly reduce blood pressure prediction errors, and introducing GRU layers to enhance SE modules can further improve the prediction accuracy and enhance the ability to extract pulse signal features.
- Building upon the improved skip-connection method, the effectiveness of replacing conventional convolution modules in the sampling paths with residual modules was verified. Model 4 and Model 5 are based on Model 2 but use ordinary residual modules and sparse residual modules for feature transmission, respectively. Compared to Model 2, the MAE for SBP predictions was reduced by 0.59 and 0.67, respectively, for Model 4 and Model 5. Similarly, for DBP predictions, the MAE was reduced by 0.17 and 0.37, respectively. This indicates that replacing the original conventional convolution modules with residual modules in both the downsampling and upsampling paths can further improve the blood pressure prediction accuracy, alleviate the gradient vanishing problem, and enhance the robustness and generalization ability of the network.
- From the experimental results, Model 6 and the proposed DSRUnet model emerged as the two best-performing models. Both models have improved SE-GRU modules embedded in the skip connection and residual connection modules for feature transmission were introduced. Among them, the DSRUnet model achieved the smallest MAE results for SBP, DBP, and MBP predictions, which were 3.36, 2.35, and 2.21, respectively. While Model 6 attained the best ME, STD, and R^2^ values for DBP and MBP predictions, considering the difficulty in SBP prediction in existing blood pressure prediction tasks, and the minor differences in DBP and MBP prediction performances (ME difference of 0.26 and 0.06, STD difference of 0.03 and 0.07, R^2^ difference of 0.005 and 0.006), this study selected the more accurate DSRUnet model for SBP prediction as the optimal model. This choice validates the excellent prediction performance and stability of the proposed SE-GRU module and sparse residual connection module.

**Figure 10:**
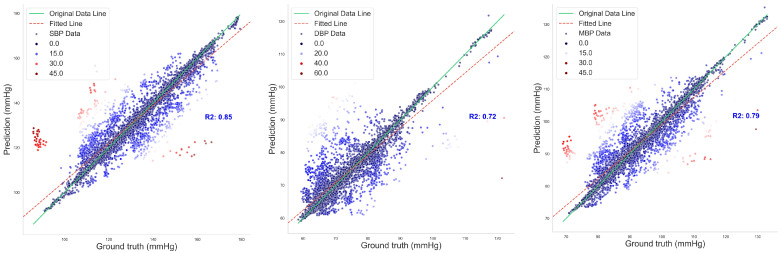
Regression fitting plot of SBP, DBP, and MBP prediction results.

Figure 10 illustrates the regression fitting results of the proposed DSRUnet model for SBP, DBP, and MBP prediction tasks. The green solid line represents the original data line, while the red dashed line represents the fitted line. Different colors denote the degree of dispersion of the data points. It can be observed that the majority of data points exhibited small errors. Additionally, the coefficients of determination (R^2^) for the SBP, DBP, and MBP prediction fitting results were 0.85, 0.72, and 0.79, respectively. From the fitting results, it can be observed that the majority of points were clustered around the line, indicating a good overall prediction performance. There were relatively few red scattered points with significant deviations.

### 4.4. Model Loss Curves and Results of Deep Supervision Monitoring

**Figure 11:**
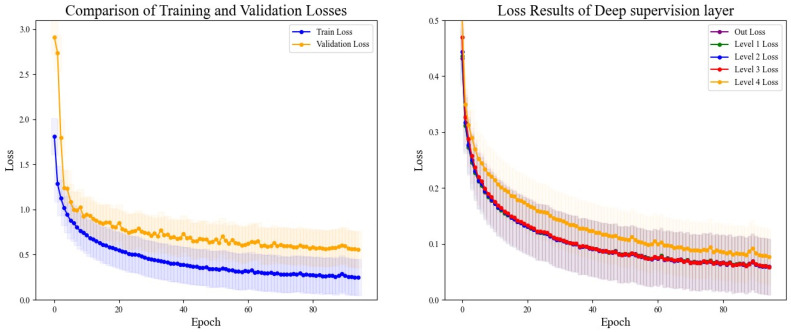
Comparison of training and validation loss results and deep supervision results. The left figure depicts the comparison of loss curves between the training and validation sets, while the right figure illustrates the comparison of loss curves from the five layers of deep supervision.

The DSRUnet model constructed in this study introduces a deep supervision mechanism to monitor the training process of the model. Five deep supervision layers were incorporated into the encoder module to learn intermediate representations and output intermediate losses for visual analysis. Additionally, to better evaluate the performance and generalization ability of the model, and to intuitively analyze the model’s performance during training and validation, the losses using the training set and validation set were recorded for a comprehensive comparison. The final results are shown in Figure 11.

From the comparative analysis, it is evident that the disparity between the training and validation losses was minimal, with the training loss consistently lower than the validation loss. Both exhibited a decreasing trend, which gradually approached a plateau, indicating that the model’s training process adheres to scientific principles without signs of overfitting or underfitting, thus providing valuable guidance. An analysis of the loss output from the five layers of deep supervision revealed higher losses during the initial stages of upsampling learning, coupled with slower descent rates, which could be alleviated through appropriate adjustments to the learning rate. Throughout all stages, the network’s learning efficacy progressively converged, with the loss values tending towards a smaller range, thus affirming the reliability of the model’s training process.

### 4.5. Comparison with Existing Methods

Comprehensively comparing the existing blood pressure prediction methods is often challenging. Different prediction methods may utilize distinct medical datasets for model training and evaluation, which could originate from diverse age groups and clinical settings with varying sampling frequencies and storage methods, resulting in significant data heterogeneity. The commonly used publicly available datasets in the field of blood pressure prediction include MIMIC-I [58], MIMIC-II [59], MIMIC-III [60], UCI-BP, and the Queensland Vital Signs Dataset [61], among others. Notably, there is a scarcity of datasets specifically tailored for blood pressure prediction tasks, with limited studies solely relying on the UCI-BP dataset, which is derived from a subset of the MIMIC-II database following preprocessing steps. Besides dataset disparities, differences in evaluation methodologies also pose challenges in comparing different methods. Various studies may opt for different evaluation metrics, and even when using the same metrics, they may employ different evaluation techniques and cross-validation strategies, leading to substantial impacts on comparison outcomes.

In light of the aforementioned considerations, in order to scientifically assess the predictive performance of the proposed DSRUnet model and determine its relative advancement compared to existing methods and models, we refer to current mainstream evaluation methodologies [62,63,64]. Specifically, we conducted a comprehensive comparison by evaluating the overall models rather than isolated parameters. We strived to select methods with similar data processing procedures and evaluation workflows for a holistic assessment. This study conducted a comprehensive comparison with existing research in three aspects:

(a) **Direct Model Comparison**: Disregarding the impact of different data preprocessing methods, we included methods utilizing the MIMIC-II and MIMIC-III datasets for comparison alongside the DSRUnet model. We directly compared the DSRUnet model with existing models based on common evaluation metrics. The final comparative results are presented in Table 8.
(b) **Innovation Assessment Against U-net Models**: The DSRUnet model proposed in this study primarily addresses the limitations of the traditional U-net model. In order to better validate the advancement of our proposed model, we considered the existing methods for blood pressure prediction based on the U-net model for a comprehensive comparison. The results are presented in Table 9.

From Table 8, it is evident that, disregarding various influencing factors, the proposed DSRUnet model attained the highest level among the analyzed models in terms of blood pressure prediction capability. The predicted absolute mean error (|ME|) and mean absolute error (MAE) values were significantly lower than those of most existing studies, indicating smaller prediction errors from the DSRUnet model. Particularly noteworthy is the marked improvement achieved in predicting SBP compared to similar models, which holds considerable significance for the task of blood pressure prediction given the historical challenge associated with predicting systolic pressure. Overall, although the DSRUnet model did not outperform some existing models on certain metrics (such as R^2^), its performance advantage remains substantial when considering multiple indicators. It is imperative to emphasize that model performance in blood pressure prediction should not rely solely on a single metric, but should encompass various factors including accuracy, stability, and clinical applicability. Furthermore, the evaluation system of the DSRUnet model is relatively comprehensive, covering the majority of evaluation metrics and possessing the capability to recover real-time blood pressure signals from single PPG signals, a feature not commonly found in other models. This attribute gives the DSRUnet model a significant advantage in practical clinical applications, as it can provide faster and more precise blood pressure predictions.

The proposed DSRUnet model demonstrated consistent performance across different metrics, with all prediction results meeting the A-grade standards outlined by the British Hypertension Society (BHS) and also aligning with the standards set by the Association for the Advancement of Medical Instrumentation (AAMI). This underscores its promising clinical applicability in blood pressure prediction.

Table 9 presents a comparative analysis of the existing methods for continuous non-invasive blood pressure prediction based on PPG signals and U-net architectures. During the comparison, it was noted that R^2^, a commonly used evaluation metric, was not calculated in these methods. Only a few studies mentioned the calculation results of the correlation coefficient (r) when performing parameter regression fitting. Therefore, we included the calculation of the correlation coefficient (r) for predicting SBP and DBP using the DSRUnet model for localized comparisons, as described in Equation (22).

(22) $$r=\frac{N\left(\sum_{i=1}^{N}y_{i}{\hat{y}}_{i}\right)-\left(\sum_{i=1}^{N}y_{i}\right)\left(\sum_{i=1}^{N}{\hat{y}}_{i}\right)}{\sqrt{\left[N\sum_{i=1}^{N}y_{i}^{2}-{\left(\sum_{i=1}^{N}y_{i}\right)}^{2}\right]\left[N\sum_{i=1}^{N}{\hat{y}}_{i}^{2}-{\left(\sum_{i=1}^{N}{\hat{y}}_{i}\right)}^{2}\right]}},$$

where N presents the number of samples, $y_{i}$ represents the true blood pressure value, and ${\hat{y}}_{i}$ represents the predicted blood pressure value.

When selecting specific comparison methods, besides considering the necessity of employing the encoder–decoder architecture of the U-net model, this study aimed to choose systems trained on similar datasets and relatively large datasets for comparison. It can be observed that the proposed method, which combines deep sparse residual U-net with improved SE skip connections, achieved superior performance compared to the majority of the U-net-based systems. Additionally, it boasts a comprehensive evaluation framework. It can accurately reconstruct blood pressure waveforms using only a single PPG signal, without the need for additional physiological signals, a feature uncommon in the existing methods. In particular, DSRUnet exhibited the lowest mean error and mean absolute error in SBP predictions, indicating a higher precision in SBP prediction compared to the existing U-net-based methods. This was achieved with only a slight compromise in DBP prediction performance. This suggests that the innovative structure of DSRUnet might be more suitable for SBP prediction, providing valuable insights for improving SBP prediction performance in the future. However, optimizing the DSRUnet structure for DBP prediction remains an area for further exploration. Furthermore, the calculated correlation coefficient (r) results of 0.92 and 0.86 demonstrate a strong correlation between the predicted and actual values, further validating the scientific rigor and credibility of the proposed method.

Combining the comparison results from both sections, it becomes evident that achieving optimization across all evaluation metrics in the field of continuous non-invasive blood pressure prediction is challenging. This is attributed to the inherent characteristics of blood pressure prediction tasks mentioned earlier. Moreover, the lack of a comprehensive prediction system that encompasses all feature datasets and measurement methods could be a potential avenue for future research. From this perspective, solely pursuing the “superiority” of data metrics while overlooking the fairness of evaluation brought about by the characteristics of blood pressure prediction tasks may not be highly persuasive.

**Figure 12:**
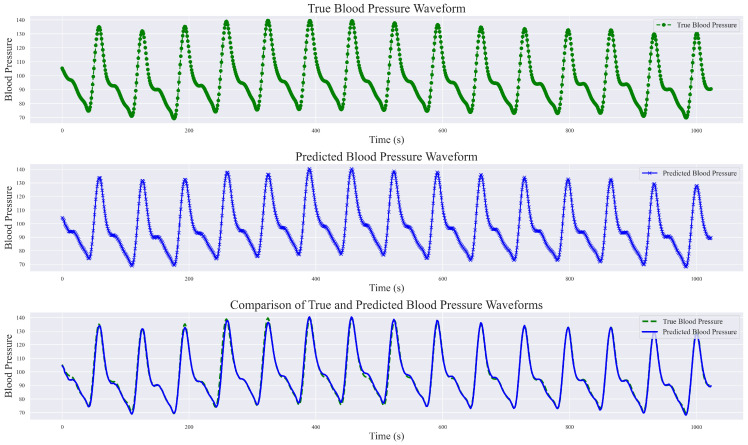
Comparison of true blood pressure waveform and predicted blood pressure waveform.

During the exploration of relevant methods, it was noted that the original input signals varied, encompassing both raw PPG signal data and raw waveform features, as well as inputs fused from multimodal information such as ECG, first-order derivatives of PPG, and second-order derivatives of PPG, among others. In this study, a singular PPG signal was employed for continuous blood pressure signal prediction. The final prediction results not only include numerical predictions but also encompass predictions of the blood pressure waveform. Analyze and present the results of a randomly selected set of sampling bands, each with a length of 1024, showcasing real blood pressure waveforms, predicted blood pressure waveforms, and a comprehensive comparison between the two, as illustrated in Figure 12.

This study demonstrated the robust performance of the DSRUnet model in estimating blood pressure waveforms by converting input PPG signals into corresponding predicted blood pressure waveforms. A comparative analysis with real blood pressure waveforms revealed that the DSRUnet model achieved a waveform prediction MAE of 3.37 mmHg, STD of 6.33 mmHg, and R^2^ value of 0.91. A visual inspection of the graphs indicated that the overall shapes, amplitudes, and trends of the predicted blood pressure waveforms closely match those of the actual blood pressure waveforms. Minor discrepancies in phase and amplitude were observed only at certain peaks, troughs, and reperfusion traces, corresponding to errors in the SBP and DBP predictions. Additionally, some variations were observed in the prediction of individual systolic phases (i.e., the stage where the arterial pressure reaches its peak), which may correspond to challenges encountered in SBP prediction. Overall, the predicted blood pressure waveforms not only closely matched the real waveforms but also accurately described the systolic and diastolic processes of blood pressure changes, capturing the corresponding peak points, trough points, and rebound traces. Moreover, the predictions were unaffected by motion artifacts in the PPG signal and alleviated the phase lag issues. Generally, ABP waveforms are collected and stored invasively. Through the proposed DSRUnet model, real-time reliable ABP waveforms can be reconstructed from PPG signals acquired using optical sensors, thus expanding the possibilities for clinical applications.

## 5. Conclusions

This study proposes a continuous non-invasive blood pressure prediction model, named DSRUnet, based on a deep sparse residual U-net combined with an improved SE skip connection. Utilizing only a single PPG signal, the model produced high-precision predictions of SBP, DBP, and MBP, as well as an accurate visualization of blood pressure waveforms. The integration of BP parameters and waveform patterns assists in identifying cardiovascular abnormalities, providing new possibilities for clinical research in hospitals and deployment studies for medical edge devices.

Specifically, the DSRUnet model employs a single PPG signal as the network input and utilizes an end-to-end U-net structure with highly symmetrical features for feature extraction. Sparse residual connection modules were introduced in the upsampling and downsampling paths to replace the ordinary convolutional modules to better capture subtle feature variations in the original PPG signal and preventing performance degradation. To model and weight global feature information more effectively, an improved SE-GRU module was embedded in the skip connections to extract the temporal features of the PPG signal through the GRU layer and enhancing the model’s generalization performance. Furthermore, a deep supervision mechanism was added to each layer’s output in the upsampling path to guide the learning of effective feature representations in the lower layers and alleviate the problem of gradient vanishing. Through ablation experiments on the UCI-BP dataset and comparisons with existing models, the effectiveness and advancement of the DSRUnet model were verified. When comparing with existing studies, we took into account the impact of data processing and evaluation procedures on the prediction outcomes. By establishing two sets of contrasting principles, we found that solely assessing system superiority based on prediction results is not objective. What is required for blood pressure prediction tasks is a comprehensive and adaptable system that remains stable across various data types. The experimental results indicated that the proposed DSRUnet model achieved higher prediction accuracy than most existing models, with a significant improvement in SBP prediction compared to the majority of the existing blood pressure prediction models. The model’s ability to accurately predict blood pressure waveforms is also relatively rare in existing research. Additionally, the model’s prediction results meet the A-grade standard of the BHS and fulfill the basic requirements of the AAMI standard, showing practical application potential in the field of intelligent wearable medical devices.

Building upon the traditional U-net model, this study balanced model complexity and prediction performance by optimizing the model structure. The proposed sparse residual connection modules and SE-GRU modules provide insights for researchers in other areas of blood pressure prediction, such as introducing models that can better extract temporal signal features, such as LSTM and GRU, to explore the underlying patterns between PPG signals and blood pressure signals, contributing to the field of blood pressure prediction.

In future work, we will delve deeper into the following issues:

1. Physiological characteristics vary from person to person, making it challenging for a single model to accurately predict blood pressure for different individuals. We will explore the mechanisms behind individual differences in blood pressure prediction tasks, considering methods such as network model optimization and transfer learning, to improve the model’s ability to generalize across individuals and resist noise interference.
2. In blood pressure prediction, the number of samples within the normal blood pressure range is often much larger than those within the high or low blood pressure ranges, resulting in dataset imbalance issues. In future research, we will address the problem of imbalance regression caused by insufficient datasets, and consider appropriate data preprocessing methods and data balancing techniques to enhance the prediction stability and accuracy of the blood pressure prediction model under data imbalance conditions.
3. The proposed DSRUnet method appears to be more focused on predicting SBP. In future research, we will consider adjusting the model structure and data processing methods to enhance the DBP prediction performance while maintaining its performance in predicting SBP. Additionally, we will explore integration with wearable devices for enhanced prediction capabilities.

## Figures and Tables

**Table 1:** Statistics of raw UCI-BP data.

	Mean (mmHg)	Std (mmHg)	Min (mmHg)	Max (mmHg)
SBP	134.19	22.93	71.56	199.99
DBP	66.14	11.45	50	165.17
MBP	90.78	14.15	59.96	176.88

**Table 2:** UCI-BP data statistics after data preprocessing.

	Mean (mmHg)	Std (mmHg)	Min (mmHg)	Max (mmHg)
SBP	134.31	17.99	85.86	179.36
DBP	72.98	9.48	57.79	134.22
MBP	93.42	9.92	69.01	139.81

**Table 3:** Model settings for comparison of different modules. “√” represents the inclusion of this module, and “―” represents the exclusion of this module.

	Deep Supervision	Skip Connection		Downsampling and Upsampling	
	DS	SE	SE-GRU	Resnet	Sparse Resnet
Model 1	√	―	―	―	―
Model 2	√	√	―	―	―
Model 3	√	―	√	―	―
Model 4	√	√	―	√	―
Model 5	√	√	―	―	√
Model 6	√	―	√	√	―
DSRUnet	√	―	√	―	√

**Table 4:** BHS standard for classification of prediction levels.

BHS Standard			
	≤5 mmHg	≤10 mmHg	≤15 mmHg
Grade A	60%	85%	95%
Grade B	50%	75%	90%
Grade C	40%	65%	85%

**Table 5:** Evaluation results of BHS standards for different models. Where SBP represents Systolic Blood Pressure, DBP represents Diastolic Blood Pressure, MBP represents Mean Arterial Pressure, and the last column of the table represents the three grades A, B, and C of the BHS standard.

Model	Task	Threshold Range			Grade
		≤5 mmHg	≤10 mmHg	≤15 mmHg	
Model 1	SBP	62.08%	80.52%	89.88%	C
	DBP	78.16%	92.83%	97.03%	A
	MBP	81.30%	94.65%	97.66%	A
Model 2	SBP	72.58%	88.16%	94.38%	B
	DBP	83.85%	94.11%	98.16%	A
	MBP	85.49%	95.04%	97.94%	A
Model 3	SBP	76.37%	89.63%	95.18%	A
	DBP	84.93%	94.86%	97.96%	A
	MBP	86.59%	94.99%	97.61%	A
Model 4	SBP	79.09%	89.96%	94.95%	A
	DBP	84.54%	94.26%	97.86%	A
	MBP	86.08%	94.85%	97.86%	A
Model 5	SBP	78.71%	89.91%	95.57%	A
	DBP	85.95%	95.34%	98.22%	A
	MBP	86.18%	94.77%	97.75%	A
Model 6	SBP	79.93%	89.63%	95.45%	A
	DBP	85.54%	94.84%	98.14%	A
	MBP	87.06%	95.38%	97.76%	A
DSRUnet	SBP	81.44%	90.38%	95.79%	A
	DBP	85.46%	94.39%	98.00%	A
	MBP	87.22%	95.07%	97.73%	A

**Table 6:** The AAMI standard evaluation results of the proposed DSRUnet model.

Model	Task	Evaluation Metrics		No. of Subjects	Pass or Not
		ME	STD		
DSRUnet	SBP	−0.15	6.71	244	Yes
	DBP	−0.54	4.54		Yes
	MBP	−0.41	4.36		―
AAMI standard	SBP/DBP	≤5	≤8	≥85	―

**Table 7:** Comparison of experimental results of evaluation indicators obtained from different models. “↓” indicates that a lower value is better, and “↑” indicates that a higher value is better.

Model	SBP (mmHg)				DBP (mmHg)				MBP (mmHg)			
	|ME| ↓	MAE ↓	STD ↓	R^2^ ↑	|ME| ↓	MAE ↓	STD ↓	R^2^ ↑	|ME| ↓	MAE ↓	STD ↓	R^2^ ↑
Model 1	1.16	6.11	9.73	0.715	3.01	3.98	5.53	0.659	1.62	3.47	5.35	0.726
Model 2	1.70	4.56	7.56	0.784	0.75	2.84	4.74	0.697	1.07	2.83	4.63	0.759
Model 3	1.27	4.16	7.45	0.818	0.47	2.60	4.72	0.703	0.73	2.57	4.66	0.764
Model 4	0.59	3.97	7.14	0.833	0.96	2.67	4.65	0.712	0.44	2.45	4.53	0.777
Model 5	1.05	3.89	7.35	0.823	0.30	2.47	4.54	0.725	0.60	2.39	4.42	0.787
Model 6	0.49	3.57	6.71	0.852	0.28	2.51	4.51	0.730	0.35	2.33	4.29	0.800
DSRUnet	0.15	3.36	6.61	0.856	0.54	2.35	4.54	0.725	−0.41	2.21	4.36	0.794

**Table 8:** Direct comparison results between the proposed DSRUnet model and existing models. The symbol “—” indicates cases where specific metrics were not computed for a particular study. In this context, A, B, C, and D represent the evaluation grades under the BHS standard. When assessing the DSRUnet model according to the AAMI standard, “P” denotes compliance with the standard, while “F” indicates failure to meet the standard.

Method	Year	Dataset	Signals	Input Type	SBP|DBP (mmHg)					
					|ME| ↓	MAE ↓	STD ↓	R^2^ ↑	BHS	AAMI
SVM [43]	2015	MIMIC-II	PPG, ABP	Raw data	―	12.38|6.34	16.17|8.45	―	D|B	―
Adaboost [65]	2019	MIMIC-II	PPG, ECG, ABP	Raw data	0.05|0.19	3.97|2.43	8.90|4.17	―	D|A	F|P
BiLSTM [66]	2020	MIMIC-II	PPG, ECG	Features	4.64|3.16	6.73|2.52	14.51|6.44	―	B|A	F|P
CNN + LSTM [67]	2020	MIMIC-II	PPG, ABP	Raw data	1.91|0.67	3.97|2.10	5.55|2.84	―	A|A	P|P
PPG2ABP [26]	2020	MIMIC-III	PPG, ABP	Raw data	1.58|1.62	5.73|3.45	10.69|6.86	―	B|A	F|P
RandomForest [68]	2020	MIMIC-II	PPG, ECG	Features	―	9.00|5.48	―	0.72|0.71	―	―
Modified U-net [25]	2021	MIMIC-III	PPG, ABP	Raw data	―	3.68|1.97	4.42|2.92	0.95|0.94	A|A	P|P
RDAE [24]	2021	MIMIC-II	PPG, ABP	Raw data	1.65|1.28	5.42|3.14	6.64|3.74	―	B|A	P|P
DeepCNAP [48]	2022	MIMIC-II	PPG, ABP	Raw data	1.23|0.53	3.40|1.75	5.40|2.81	0.93|0.90	A|A	P|P
CycleGAN [69]	2022	MIMIC-II	PPG, ABP	Raw data	―	2.89|3.22	4.52|4.67	―	A|A	—
ARIU [70]	2022	MIMIC-III	PPG, ABP	Raw data	―	4.75|2.81	6.72|4.59	―	A|A	P|P
TFNet-MTD2L [46]	2023	MIMIC-II	PPG, ABP	Raw data	0.48|0.39	5.89|3.35	8.93|5.08	0.61|0.51	B|A	F|P
UTransBPNet [71]	2023	MIMIC-II	PPG, ECG, ABP	Raw data	0.40|0.11	4.38|2.25	6.21|3.10	―	―	P|P
Ours: DSRUnet	2024	MIMIC-II	PPG, ABP	Raw data	0.15|0.54	3.36|2.35	6.61|4.54	0.86|0.73	A|A	P|P

**Table 9:** Comparison results of blood pressure prediction models based on the U-net model. ‘—’ denotes instances where a particular study did not calculate a specific metric. “↓” signifies that lower values indicate a better performance, while “↑” indicates that higher values indicate a better performance.

Method	Parameter Results of Blood Pressure Prediction Based on U-Net Model									
	SBP (mmHg)					DBP (mmHg)				
	|ME| ↓	MAE ↓	STD ↓	R^2^ ↑	r ↑	|ME| ↓	MAE ↓	STD ↓	R^2^ ↑	r ↑
BP-Net [21]	0.23	5.16	8.50	―	―	0.59	2.89	4.78	―	―
ARIU [70]	―	4.75	6.72	―	0.93	―	2.81	4.59	―	0.91
U-Net4 [72]	3.35	15.21	18.99	―	0.49	0.21	7.12	9.38	―	0.39
MAGU [73]	0.21	3.49	5.40	―	―	0.43	2.11	3.24	―	―
DEU-Net [29]	0.42	3.80	6.86	―	―	0.03	1.81	4.52	―	―
iPPG2BP [74]	1.51	6.73	9.22	―	―	1.00	5.10	6.78	―	―
Ours: DSRUnet	0.15	3.36	6.61	0.86	0.92	0.54	2.35	4.54	0.73	0.86

## Data Availability

The publicly available dataset from the UCI Machine Learning Repository was utilized in this study. This data can be found here: https://archive.ics.uci.edu/dataset/340/cuff+less+blood+pressure+estimation (accessed on 10 March 2024).
